# The Mechanism of Long Non-coding RNA MEG3 for Neurons Apoptosis Caused by Hypoxia: Mediated by miR-181b-12/15-LOX Signaling Pathway

**DOI:** 10.3389/fncel.2016.00201

**Published:** 2016-09-02

**Authors:** Xiaomin Liu, Lijing Hou, Weiwei Huang, Yuan Gao, Xin Lv, Jiyou Tang

**Affiliations:** ^1^Department of Neurology, Qianfoshan Hospital, Shandong UniversityJinan, China; ^2^Department of Pharmacological Laboratory, Shandong Academy of Chinese MedicineJinan, China

**Keywords:** MEG3, miR-181b, 12/15-LOX, infarct, MCAO

## Abstract

**Objective:** lncRNAs are recently thought to play a significant role in cellular homeostasis during pathological process of diseases by competing inhibiting miRNA function. The aim of present study was to assess the function of long non-coding RNA (lncRNA) MEG3 and its functional interaction with microRNA-181b in cerebral ischemic infarct of mice and hypoxia-induced neurons apoptosis.

**Methods:** To address this question, we performed the experiments with *in vivo* middle cerebral artery occlusion (MCAO) mice model and *in vitro* oxygen-glucose deprivation (OGD)-cultured neuronal HT22 cell line. Relative expression of MEG3, miR-181b, and 12/15-LOX (lipoxygenase) mRNA was determined using quantitative RT-PCR. Western blot was used to evaluate 12/15-LOX protein expression. TUNEL assay was performed to assess cell apoptosis.

**Results:** In both MCAO mice and OGD-cultured HT22 cell, ischemia, or hypoxia treatment results in a time-dependent increase in MEG3 and 12/15-LOX expression and decrease in miR-181b expression. Knockdown of MEG3 contributes to attenuation of hypoxia-induced apoptosis of HT22 cell. Also, expression level of MEG3 negatively correlated with miR-181b expression and positively correlated with 12/15-LOX expression. In contrary to MEG3, miR-181b overexpression attenuated hypoxia-induced HT22 cell apoptosis, as well as suppressed hypoxia-induced increase in 12/15-LOX expression. By luciferase reporter assay, we concluded that miR-181b directly binds to 12/15-LOX 3′-UTR, thereby negatively regulates 12/15-LOX expression.

**Conclusion:** Our data suggested that long non-coding RNA MEG3 functions as a competing endogenous RNA for miR-181b to regulate 12/15-LOX expression in middle cerebral artery occlusion-induced ischemic infarct of brain nerve cells.

## Introduction

In recent years, with the increasing incidence of cerebrovascular disease, the nerve injury caused by acute cerebral ischemia brings an increasingly heavy burden on individuals and society. This situation makes it a central issue to elucidate the molecular mechanisms of acute cerebral ischemia-related neuronal injury (Chen et al., 2014).

According to encode project, ~80% of the human genome is transcribed to RNA, of which ~90% genome transcribes into non-coding RNA. These non-coding RNAs are increasing recognized and defined as endogenous regulatory RNA involved in maintenance of cellular homeostasis during growth, development and pathogenesis disease. Basing on different sizes, these non-coding RNA are classified into miRNA (microRNA, 20~22 nucleotides in length) and lncRNA (long non-coding RNA, >200 nucleotides in length). Interestingly, lncRNA is recently found to be a competing endogenous RNA for miRNA and these interactions have been found in various pathological mechanisms (Tay et al., 2014).

Studies have shown that ischemia alters cerebral miRNA and lncRNAs that control epigenetic silencing or activation in mammal (Dharap et al., 2009, 2013; Zhang et al., 2015), including long non-coding RNAs (LncRNAs), also undergo changes in the poststroke brain (Mehta et al., 2015). Although no direct evidence focusing on the involvement of MEG3 in cerebral ischemic injury, it was genetic phenotype silenced in brain disease and has been recognized as a novel potentially tumor-suppressing RNA in meningioma and glioma cell (Wang et al., 2012; Ouyang et al., 2014). Moreover, several studies have suggested that miRNAs are altered in response to ischemia reperfusion injury and regulate the expression of various key elements in cell survival and apoptosis. MiR-181b is a multifunction miRNA in brain. Fox example, it can be function as a NMDA receptor dependent plasticity-responsible miRNA in hippocampal neurons (van Spronsen et al., 2013). Moreover, miR-181 is showed to be involved in synaptic plasticity and memory processing of Alzheimer's disease mice via targeting functional gene expression (Rodriguez-Ortiz et al., 2014). As for brain ischemia, miR-181 contributes to astrocyte, a neuroprotective cell for brain ischemia (Ouyang et al., 2014), cell apoptosis in ischemic injury brain and *in vitro* glucose deprivation cultured astrocyte (Ouyang et al., 2012). Moreover, downregulation of miR-181b can protect middle cerebral artery occlusion (MCAO)-induced ischemic injury of mice brain (Peng et al., 2013). To be noted, study has pointed out that MEG3 serves as a competing endogenous RNA for miR-181 in other disease model. Therefore, our present study was to evaluate the functional interaction between MEG3 and miR-181b in cerebral ischemic infract and in hypoxia-induced neuron apoptosis.

Recent studies showed that the neuronal 12/15-LOX was robustly activated in the injured brain and mediated oxidative stress-induced neuronal dysfunction contributing to neuronal death after cerebral ischemia (Han et al., 2015; Jung et al., 2015). We currently evaluated whether ischemia-responsive lncRNA interact with miR-181b, associated with genetic phenotype change of 12/415-LOX in cerebral ischemic mice.

## Materials and methods

### Animals model of middle cerebral artery occlusion

Middle cerebral artery occlusion (MCAO) model was established in 6 month BALB/c mice. Briefly, animals were anesthetized with sodium pentobarbital (30 mg/kg) via intraperitoneal injection and were placed on heating panel inserting with rectal probe to keep 37°C temperature during operation. For the right MCAO surgical procedure, a 1 cm incision was made for exposing the right common carotid artery, external carotid artery, and internal carotid artery. A 4/0 monofilament nylon suture with a rounded tip obtained by heating was inserted into the right external carotid artery and gently advanced into the internal carotid artery until the rounded tip blocked the origin of the middle cerebral artery. Sham-operated animals underwent the same surgical operation without insertion of monofilament nylon. At 6, 12, and 24 h after the onset of permanent occlusion, animals (*n* = 6 per group) were sacrificed and, the brains were immediately removed and coronally sectioned from −1.0 to +3.0 mm bregma. These brain cortex tissues were collected and stored in −80°C refrigerator for subsequent analysis of RNA and protein determination. This study was approved by the Ethical Committee of Shandong University.

To evaluate role of MEG3 in cerebral ischemia injury, lipid nanoparticles-formulated si- MEG3 or si-control (2.5 mg/kg body weight) was intravenously injected into mice before MCAO operation. One day post injection, MCAO mice model was established. At 24 h after operation, motion function of mice was assessed on the basis of moving distance within 3 min. Animals were then sacrificed for evaluation of edema volume and infarct volume.

### Quantification of infarct volume and edema volume

Animals were sacrificed and the brain was removed. The brain cortex tissues were made into brain slices and used for analysis of infarct volume and edema volume. Coronal sections of brain (30 μm; separated by ~420 μm) were cut and then stained with 0.1% thionin. The infract area and edema area were analyzed and calculated with ImageJ software. Infarct volume and brain edema were determined by integrating the infarct area of different brain slices areas with the use of cylinder and cone rules. To avoid subjective factors, investigator who performs the analysis was blinded to the treatment groups.

### HT22 cell culture and oxygen glucose deprivation (OGD)

HT22 cells (a mouse hippocampal cell line) were obtained from the American Type Culture Collection (ATCC). Cells were cultured in Dulbecco's Modified Eagle's Medium (DMEM) containing 10% fetal bovine serum (FBS, Gibco) with 2 mM glutamine, 100 U/mL penicillin, and 100 μg/mL streptomycin and maintained at 37°C in a humidified incubator with 5% CO_2_. Medium was replaced every 2 days. For *in vitro* OGD model, HT22 cells were pre-exposed to an oxygen-depleted, glucose-free medium for 15 min and then incubated in a hypoxic chamber (under condition of 5% CO_2_/95% N_2_) at 37°C. The hypoxic time was set up at three intervals of 6, 12, and 24 h respectively.

### Quantitative RT-PCR

Total RNA were extracted with TRIzol reagent, and miRNAs were extracted by TRIzol SM Reagent (HaiGene, Haerbing, China). After quantified by spectrophotometer, equal RNA of each sample was reversely transcribed into cDNA using PrimeScript® miRNA or mRNA cDNA synthesis kit (TaKaRa, Tokyo, Japan). The generated cDNA was amplified using Platinum SYBR Green qPCR Super Mix UDC (Invitrogen, Carlsbad, CA) in an ABI Prism 7000 (Applied Biosystems, Foster City, CA). The genes encoding mouse MEG3, 12/15-LOX mRNA, miR-181b were amplified with the following primer pairs. Relative expression of these genes was calculated by the 2^−ΔΔCT^ value method. Relative expression of MEG3 and 12/15-LOX mRNA was normalized to GAPDH, and relative expression of miR-181b was normalized to U6.

Primers for MEG3, 12/15-LOX mRNA, miR-181b are as follows:

MEG3: 5′-CTGCCCATCTACACCTCACG-3′ (sense) and 5′-CTCTCCGCCGTCTGCG CTAGGGGCT-3′ (antisense)
12/15-LOX: 5′-ACCCCACCGCCGATTTT-3′ (sense) and 5′- AGCTTCGGACCCAGCATTT-3′ (antisense)
miR-181b: 5′-CAGACATCTCTGCCTCACA-3′(sense) and 5′-TTGCGGTTCTGTCTTCAGC-3′ (antisense)

### Western blot

Cells were lysed in RIPA buffer containing mixture proteinase inhibitor. Proteins were extracted from lysate by centrifugation at 10,000 × g for 10 min at 4°C. Total protein concentration was measured and normalized with the BCA protein assay kit (Pierce Biotechnology, IL, USA). Twenty-five Microgram of total protein was loaded in sodium dodecyl sulfate polyacrylamide gel electrophoresis (SDS-PAGE) and were migrated by electrophoresis. Proteins in gel were then transferred to polyvinylidene fluoride membranes (Millipore). After blocked by 5% non-fat milk in Tris-buffered saline containing 0.1% Tween 20 (TBS-T) for 2 h at room temperature, membrane was incubated with antibodies against 12/15-LOX and β-actin at 4°C overnight. The following day, membrane was washed twice with TBST and was incubated with goat anti-rabbit IgG-HRP (1:4000) for 2 h at room temperature. Bands were detected by chemiluminescence ECL kit in X-ray film developer and quantified using ImageJ software.

### TUNEL assay

TUNEL assay is a method to detect fragmented DNA of apoptotic cell and was used to evaluate cultured HT22 cells apoptosis with Click-iT® Plus TUNEL assay for *in situ* Apoptosis Detection (Thermo Fisher Scientific) according to the manuals. Collected cells were washed by PBS, and re-suspended at a concentration of 10^7^/ml in PBS. One hundred microliter of cell suspension was then cultured in 48-well plate. After fixed by addition of 2% formaldehyde in PBS, cells were collected and postfixed with 70% ice-cold ethanol. The fixed cell were added with TUNEL reaction buffer and incubated in TUNEL reaction mixture in dark for 60 min at 37°C. After reaction, samples were subjected to fluorescence microscope (Olympus, Tokyo, Japan) and eight visual fields of them were randomly selected to count the cells with positive signals.

### Transfection

For siRNA knockdown of MEG3, HT22 cells were transfected with MEG3 siRNA (si-MEG3) or non-targeting control siRNA. For miR-181b knockdown, HT22 cells were transfected with MISSION® Synthetic microRNA Inhibitor (Sigma-Aldrich) for miR-181b with negative control (NC) as control.

For overexpressing MEG3, recombinant pcDNA plasmid of MEG3 gene was transfected into HT22 cells with pcDNA empty plasmid as control. For miR-181b overexpression, the cells were transfected with miRIDIAN Mimics for miR-181b (Dharmacon) as compared with pre-negative control (pre-NC). All transfections were carried out using lipofectamine 2000 according to manufacturer guidelines and cells were harvested after 48 h for subsequent experiments.

### Dual luciferase reporter assay for 12/15-LOX promoter activity

The promoter region of the 12/15-LOX gene was amplified from mouse genomic DNA and was then introduced into the KpnI and BglII sites of pGL3-Basic (Promega, Madison, WI) to generate 12/15-LOX 3′-UTR-WT. Mutate 12/15-LOX gene was also amplified and introduced into pGL3-Basic to generate 12/15-LOX 3′-UTR-MU (served as control). The resulting plasmid was designated as pGL3-12/15-LOX. HT22 cells were co-transfected with miR-181b mimic or miR-181b inhibitor or negative control and pGL3-12/15-LOX luciferase construct. Cells were harvested at 24 h post-transfection for luciferase activity analysis using the Dual-Luciferase Reporter Assay system (Promega, Madison, WI). The amount of firefly luciferase activity was presented as the increase or decrease (n-fold) relative to the value for the sample lacking the miR-181b or overexpressing miR-181b.

### Statistical analysis

Data were presented as means ± *sd* (standard deviation). Results were analyzed either by one-way ANOVA followed by Dunnett's multiple comparison tests or paired *t* test (version 5.00; GraphPad Prism Software Inc., San Diego, CA). Statistical significance was considered for *P* < 0.05.

## Results

### Gene phenotype changes of infarction tissues in middle cerebral artery occlusion mice and in neuronal HT22 cell during glucose deprivation

**Figure 1:**
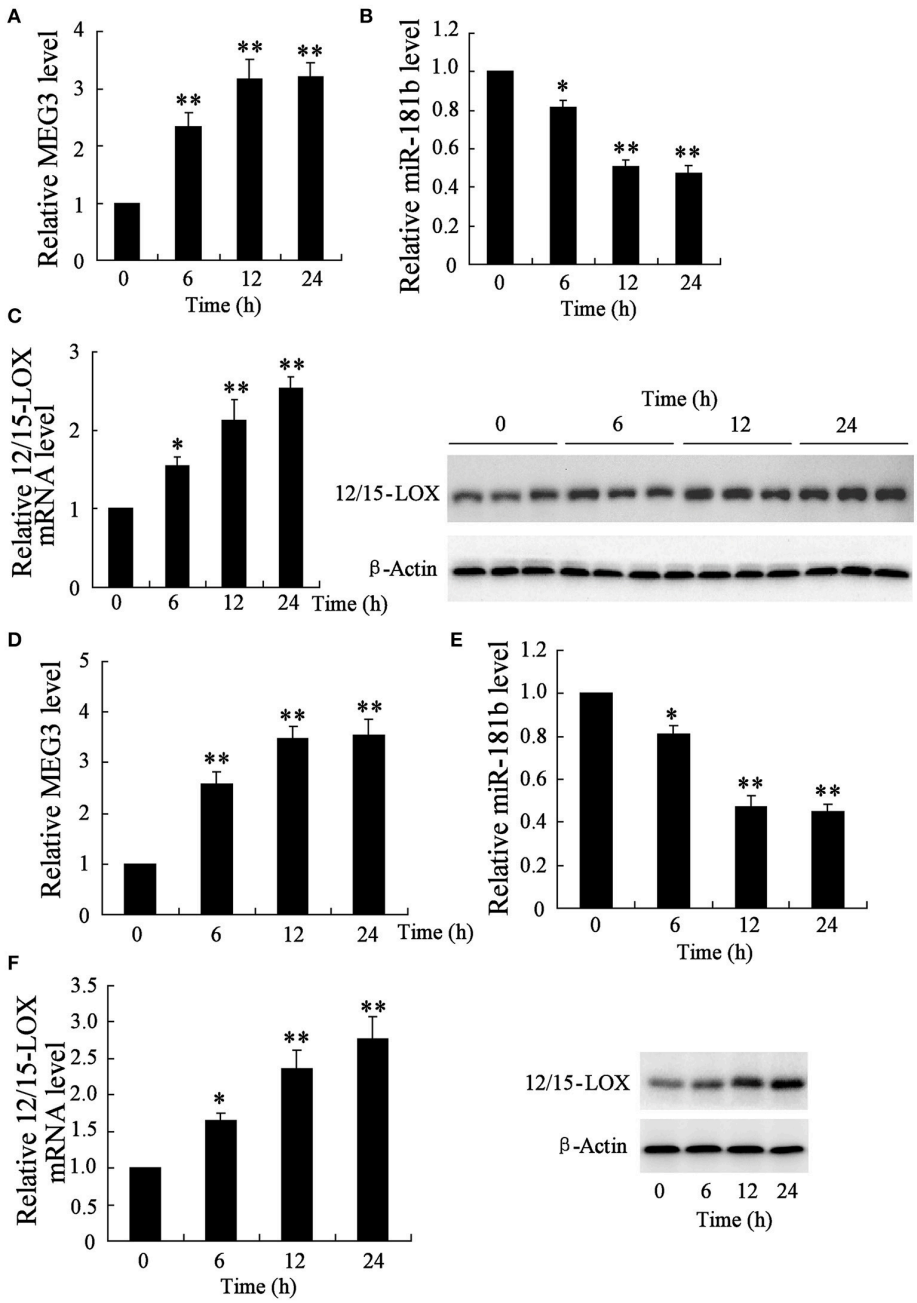
**Time course of genes expression in infarction area of middle cerebral artery occlusion mice and in hypoxia-stimulated HT22 neural cell line**. After a series of 6, 12, and 24 h animal establishment, quantitative RT-PCR was performed to determine the relative expression of **(A)** MEG3, **(B)** miR-181b, and **(C)** 12/15-LOX mRNA; **(C)** representative blots of 12/15-LOX protein expression by Western blot. HT22 cells were experienced 6, 12, and 24 h of glucose deprivation for following gene expressional analysis. Relative expression of **(D)** MEG, **(B)** miR-181b, and **(E)** 12/15-LOX, and **(F)** 12/15-LOX protein expression were determined. Data are presented as mean ± *sd*. ^*^P < 0.05, ^**^P < 0.01 compared with sham with no MCAO surgery or cell with no treatment.

To examine aberrant genes expression in response to ischemia, mice middle cerebral artery occlusion model was established and infarction tissues were isolated for subsequent analysis of quantitative RT-PCR and western blot. The data showed that, at 6, 12, and 24 h post model establishment, relative expression level of MEG3 is increased in a time-dependent manner (Figure 1A), whereas miR-181b is time-dependently downregulated (Figure 1B). Specifically, ischemia time-dependently caused upregulation of 12/15-LOX mRNA and protein (Figure 1C).

To determine those abnormal genes expression in hypoxia-injured neural cell, neuronal HT22 cell-Oxygen-glucose deprivation (OGD) *in vivo* model of brain hypoxia was established (Tasca et al., 2015). The data showed that OGD treatment causes the same gene expression trend of neuronal cell as MCAO mice, in which showed upregulation of MEG3 (Figure 1D), downregulation of miR-181b (Figure 1E), and upregulation of 12/15-LOX in a time-dependent manner (Figure 1F).

### Manipulation of MEG3 affects hypoxia-induced HT22 cell apoptosis

**Figure 2:**
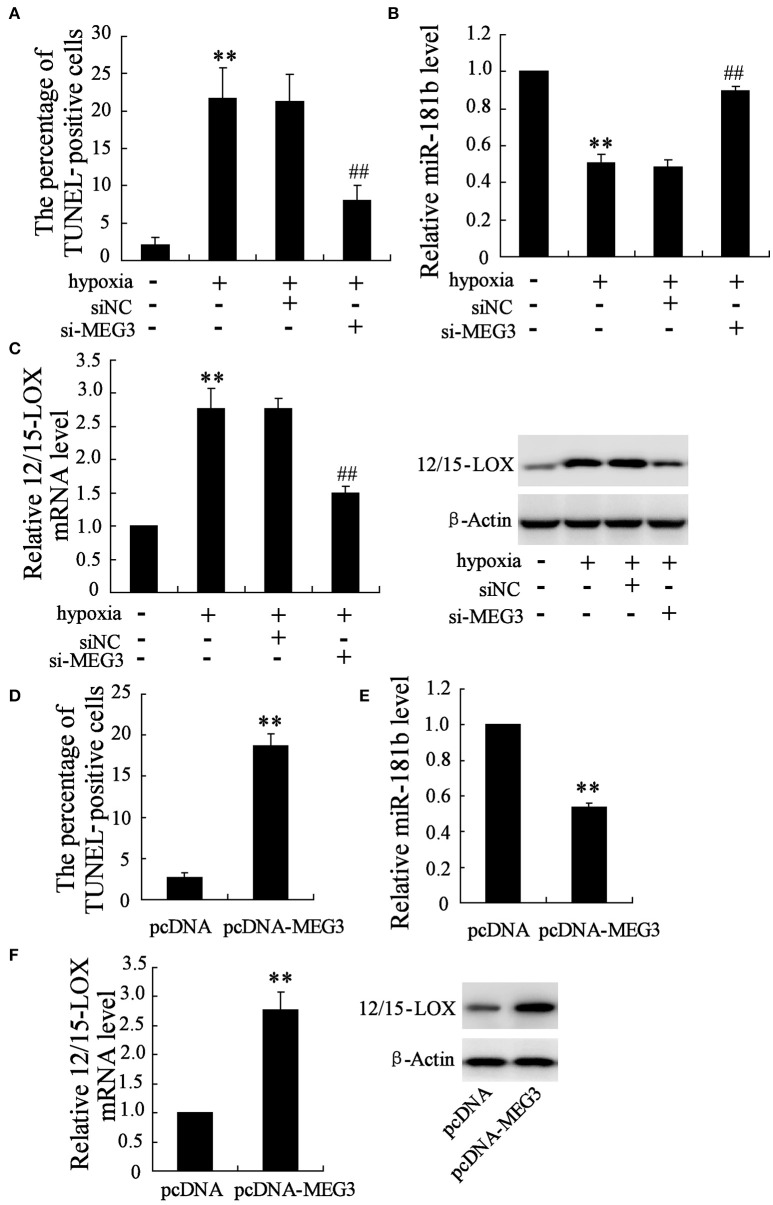
**Manipulation of MEG3 affects hypoxia-induced apoptosis in HT22 cell**. HT22 cells were pre-transfected with si-MEG3 or si-NC for 24 h and then deprived 12 h of glucose-free DMEM. **(A)** Cell apoptosis was evaluated by TUNEL assay. Expression level of **(B)** miR-181b and **(C)** 12/15-LOX mRNA and protein were determined. Also cells were transfected with pcDNA-MEG3 or pcDNA empty plasmid for 24 h. **(D)** Cell apoptosis, expression level of **(E)** miR-181b and **(F)** 12/15-LOX mRNA and protein were evaluated. Data are presented as mean ± *sd*. ^**^P < 0.01 compared to cell with no hypoxia treatment or pcDNA; ^##^*P* < 0.01 compared with hypoxia +si-NC.

Considering the positive expression of MEG3 in neuronal cell responding to hypoxia, we next manipulate cellular MEG3 by RNA interference and examine the involvement of MEG3 in hypoxia-induced neuronal cell apoptosis. As shown in Figure 2A, si-MEG3 transfection significantly attenuates hypoxia-induced cell apoptosis as compared with si-negative control treatment. In particular, hypoxia-stimulated downregulation of miR-181b and upregulation of 12/15-LOX was partly recovered (Figures 2B,C).

Recombinant that containing MEG3 gene was further employed to ensure the important role of MEG3 in neuronal cell apoptosis. Under the condition of the pcDNA-MEG3 transfection, cell apoptosis percentage is up to 7.2 times than pcDNA control treatment (Figure 2D). Accordingly, MEG3 overexpression brings about inhibitory expression of miR-181b (Figure 2E) and enhances expression of 12/15-LOX mRNA and protein (Figure 2F).

### Functional interaction of MEG3 and miR-181b affects hypoxia-induced HT22 cell apoptosis

**Figure 3:**
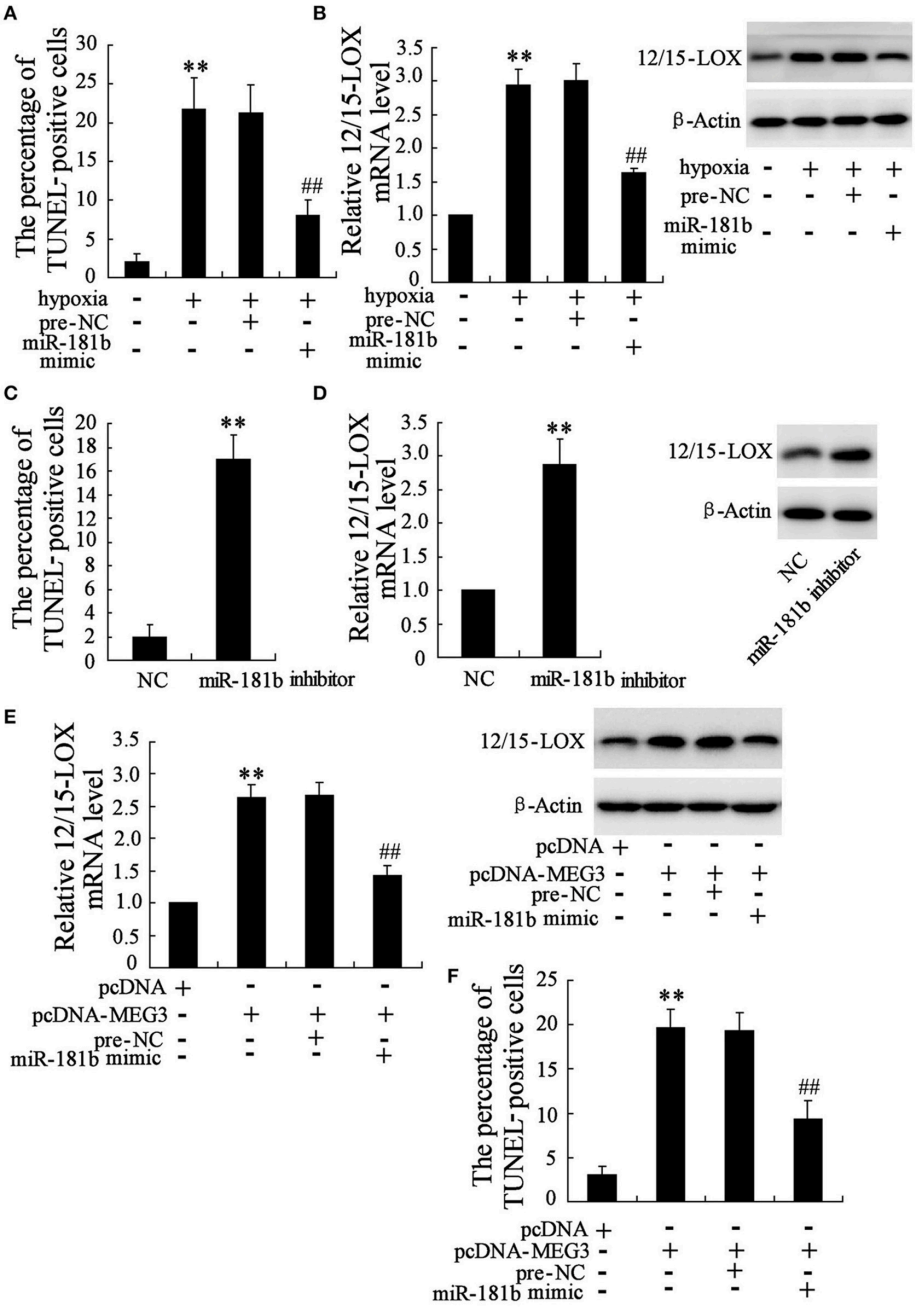
**Functional interaction of MEG3 and miR-181b mediates HT22 cell apoptosis**. HT22 cells were pre-treated with miR-181b mimic or pre-NC before exposed to glucose deprivation. **(A)** Cell apoptosis and **(B)** expression level of 12/15-LOX mRNA and protein were determined. **(C)** Cell apoptosis and **(D)** 12/15-LOX expression were analyzed in miR-181b inhibitor treated HT22 cells. Cells were then co-treated with pcDNA-MEG3 and miR-181b mimic. **(E)** Expression level of 12/15-LOX and **(F)** cell apoptosis were determined. Data are presented as mean ± *sd*. ^**^P < 0.01 compared to cell with no hypoxia treatment or NC or pcDNA; ^##^*P* < 0.01 compared with hypoxia +pre-NC or pcDNA-MEG3+pre-NC.

Considering the establishment that downregulation of miR-181b of neuronal cell in response to hypoxia, the exploration to determine whether miR-181b may also play a role in the progress of hypoxia-induced neuronal cell apoptosis is particularly important. We analyzed the cell apoptosis in miR-181b mimic transfected cells in Figure 3A, where showed that miR-181b overexpression significantly reduced cell apoptosis percentage as compared with pre-negative control treatment. Specially, 12/15-LOX is consequently downregulated by miR-181b overexpression under hypoxia condition (Figure 3B). On the contrary, RNA interference of miR-181b by miR-181b inhibitor culture under normal culture conditions effectively promotes expressions of HT22 cell apoptosis (Figure 3C) and 12/15-LOX (Figure 3D).

As indicated in above data, both MEG3 and miR-181b function as the key regulators for hypoxia-induced HT22 cell apoptosis. We therefore evaluate the functional interaction between MEG3 and miR-181b in HT22 cells by cell co-transfection with pcDNA-MEG3 and miR-181b. We observed that co-overexpression of MEG3 and miR-181b contributes to inhibitory effect of MEG3 on 12/15-LOX expression (Figure 3E), as well as abrogates pro-apoptosis action of MEG3 (Figure 3F).

### miR-181b regulates 12/15-LOX expression in HT22 cell

**Figure 4:**
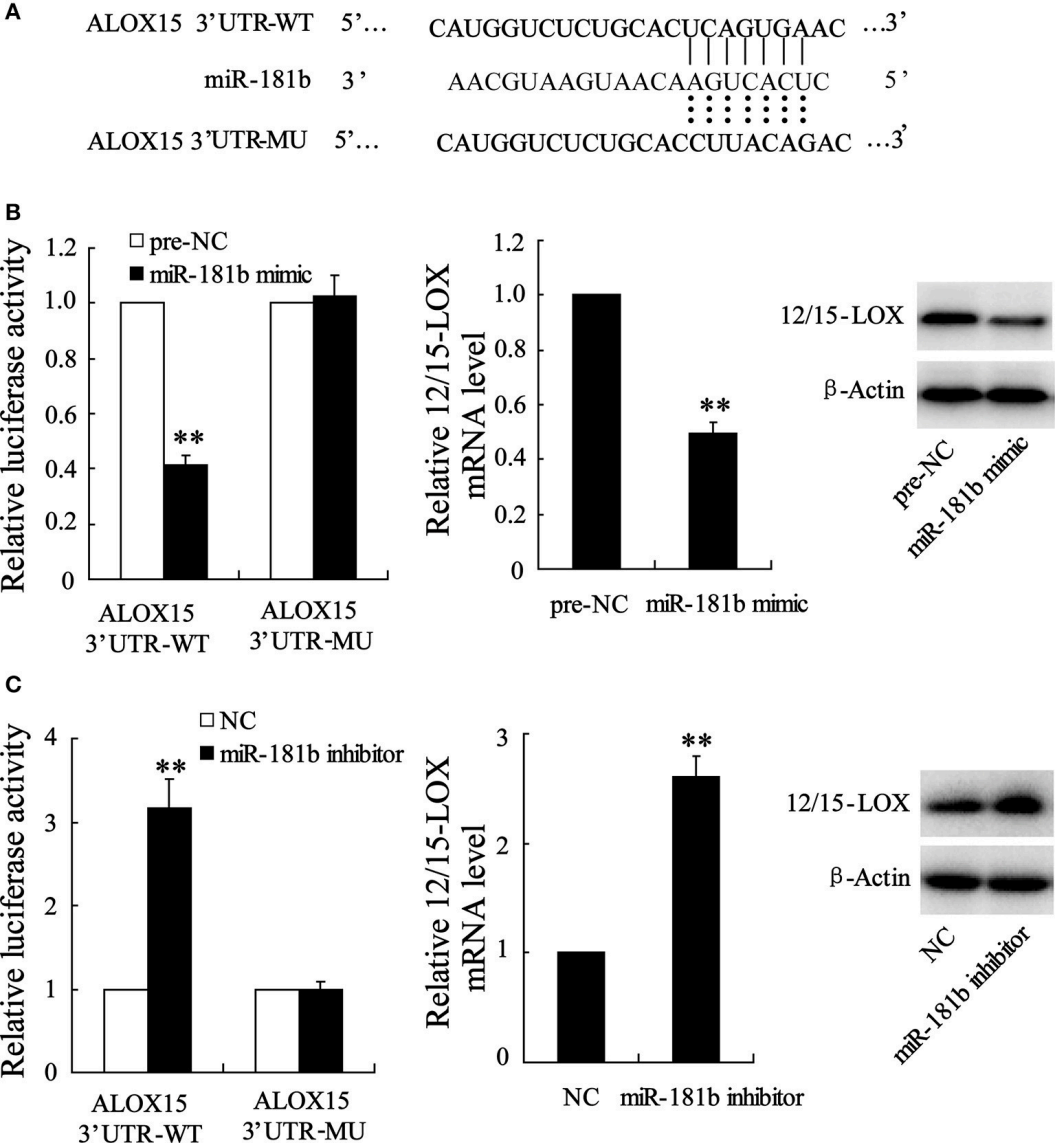
**Manipulation of cellular miR-181b regulates12/15-LOX expression in HT22 cell. (A)** Representative base-pairs between 12/15-LOX 3′-UTR-WT and miR-181b with 12/15-LOX 3′-UTR-MU as positive control. **(B)** Cells were co-treated with 12/15-LOX 3′-UTR-WT or 12/15-LOX 3′-UTR-WT and miR-181b mimic; fluorescence activity and 12/15-LOX expression were determined. **(C)** Fluorescence activity and 12/15-LOX expression were determined in 12/15-LOX 3′-UTR-WT or 12/15-LOX 3′-UTR-WT and miR-181b mimic co-transfected HT22 cells. Data are presented as mean ± *sd*. ^**^P < 0.01 compared with 2/15-LOX 3′-UTR-WT or NC.

Based on the above data suggesting a link between abnormal miR-181b level and 12/15-LOX expression, we further test whether miR-181b manipulation by siRNA knockdown or recombination plasmid transfection may directly regulate 12/15-LOX expression. By predicating in bioinformatics software, we observed that complementary sequences of miR-181b in 12/15-LOX 3′-UTR (Figure 4A). We next co-transfect 12/15-LOX 3′-UTR-WT with miR-181b mimic for purpose of evaluating transcriptional activity of 12/15-LOX. Cell with 12/15-LOX 3′-UTR-MU co-transfection was as positive control. Figure 4B showed that fluorescence activity of 12/15-LOX is reduced by miR-181b overexpression; accordingly, relative expression of 12/15-LOX is suppressed. We further investigated the effect miR-181b inhibitor on 12/15-LOX transcriptional activity and expression in Figure 4C, which showed knock down of miR-181b contributes enhancement of fluorescence activity of 12/15-LOX, as well as promotes 12/15-LOX expression (Figure 4C).

### Knock down of MEG3 improves infarct of MACO mice

**Figure 5:**
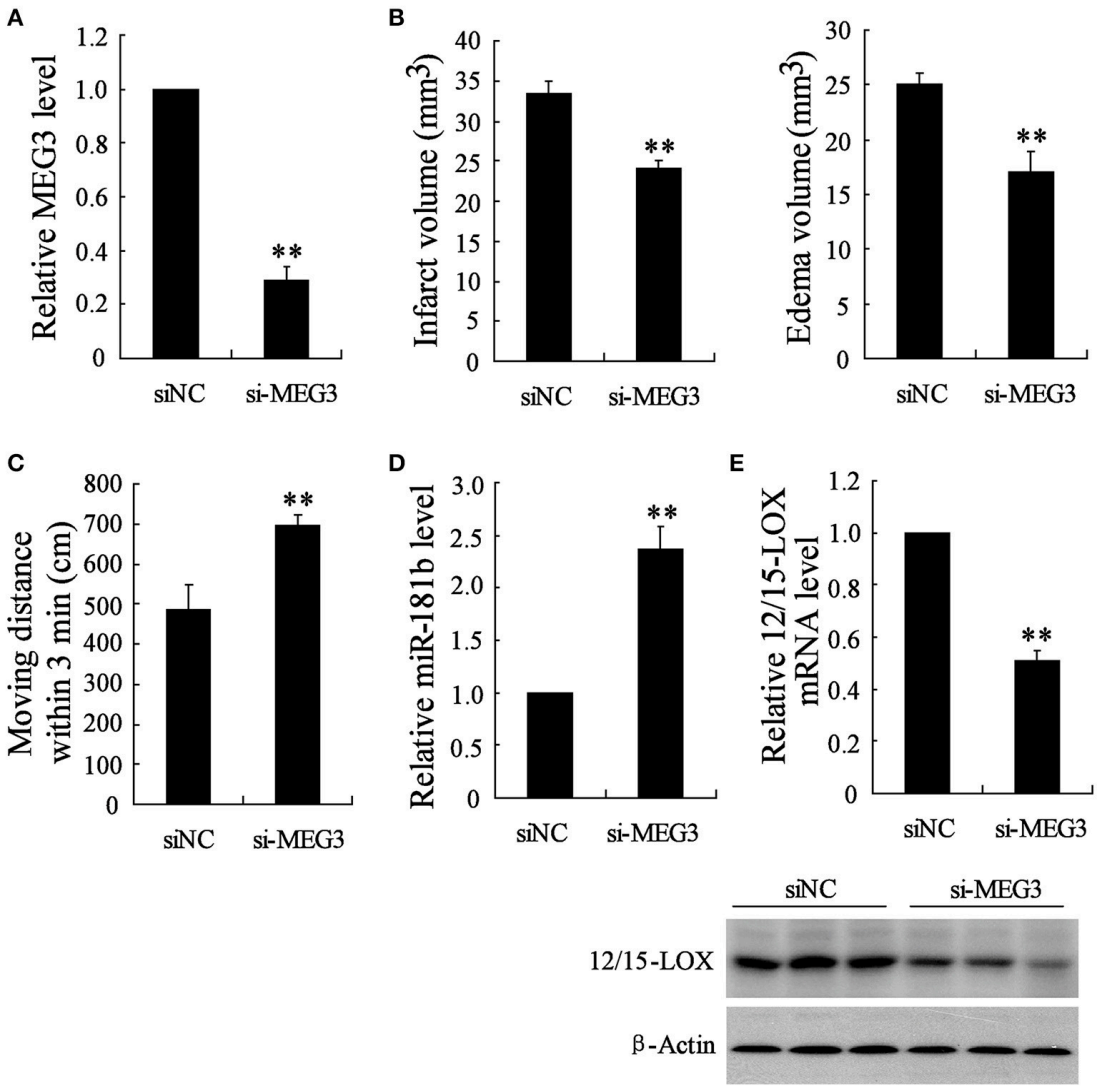
**si-MEG3 injection improves cerebral infarction**. Mice were injected with lipid nanoparticles-formulated si-control or si- MEG3 for 24 h before MCAO establishment. **(A)** Relative expression of MEG3 in infract position was determined. **(B)** Infarct volume and edama volume and **(C)** motion function were evaluated. Examination of **(D)** relative expression of miR-181b and **(E)** 12/15-LOX in infract site. Data are presented as mean ± *sd*. ^**^P < 0.01 compared with siNC.

Above data suggested that MEG3 is responsible for hypoxia-induced neuronal cell apoptosis. We next determined the pivotal role of MEG3 in cerebral infarction of MACO mice by animals injected with si-MEG3. We observed the downregulation of MEG3 in animals with si-MEG3 injection (Figure 5A). These si-MEG3 injections significantly improved MACO-induced brains infract volume and edema volume (Figure 5B) and also promoted motion function of animals (Figure 5C). To confirm the downstream miR-181b/12/15-LOX pathway of MEG3, we next determined miR-181b and 12/15-LOX in infract position. The data showed that miR-181b was upregulated and expression of 12/15-LOX mRNA and protein was suppressed in animals with si-MEG3 injection compared with si-NC (Figures 5D,E).

## Discussion

lncRNAs in the mammalian genome are characterized by evolutionary conservation and tissue-specific expression pattern. Moreover, accumulated studies have suggested that ectopic expression and/or functionality of lncRNAs play a role in various pathological processes including inflammation, oxidative stress response, and ischemic injury. A major function attributed to lncRNA is to competitively inhibit miRNA function by indirectly liberating miRNA-restrained functional protein. Therefore, our present study evaluated the interacting epigenetic change of lncRNA MEG3 to miR-181b in focal cerebral ischemia injury of mice subjected to middle cerebral artery occlusion (MCAO).

Our data described a global time dependent analysis of the levels of hippocampal MEG3 and miR-181b during cerebral ischemia and in OGD-cultured hippocampal HT22 cell line in periods of 6, 12, and 24 h post hypoxia condition. We observed that the MEG3 is gradually upregulated, whereas miR-181b is gradually downregulated in both brain and HT22 cell with increasing time of hypoxia. This data suggested the expressional negative correlation between MEG3 and miR-181b in response to brain hypoxia. Previously, the MEG3 and miR-181b works have been confirmed to be involved in cerebral disease. For example, MEG3 is recognized as a novel tumor suppressor for brain tumor (Balik et al., 2013). MEG3 is also showed to be involved in production of gonadotropin-releasing hormone in HT22 cell (Tao et al., 2015). In addition, accumulated studies have demonstrated that miR-181b plays multifunctional roles in brain disease. MiR-181 suppressed proliferation, migration, and invasion of astrocytoma and was proved to be involved in neuroinflammatory responses of astrocytes (Hutchison et al., 2013; Zhi et al., 2014). Moreover, downregulation of miR-181b can protect middle cerebral artery occlusion (MCAO)-induced ischemic injury of mice brain (Peng et al., 2013). In present study, we were the first to determine the functional interaction between MEG3 and miR-181b in cerebral hypoxia injury.

Interestingly, MEG3 has been recognized as a competing endogenous RNA for miR-181 in other experiments (Peng et al., 2015). Basing on our data and online reference, we proposed that MEG3 might function as a regulator for miR-181 in ischemic neuron. Results that support this proposal are that miR-181b is upregulated with the MEG3 knockdown under hypoxia condition; and in normal condition, expression of miR-181b is suppressed by MEG3 overexpression. Transient cerebral ischemia during MCAO surgery may cause metabolic stress or neurological disorders; while the visible appearance is death of neuron (Sun et al., 2013; Zheng et al., 2015). To understand the implication of altered lncRNA MEG3 and miR-181b in post-stoke pathophysiology of neuron survival, we currently evaluate whether this interaction promotes the functional association between MEG3/miR-181b and the hypoxia-induced neuronal cell apoptosis. The present data showed that knockdown of MEG3 or miR-181b overexpression can separately attenuate hypoxia-caused HT22 cell apoptosis. This data suggested the critical role of MEG3 and miR-181b in the development of ischemic apoptosis of neuron. Moreover, the critical role of MEG3 in cerebral ischemic injury was confirmed by decrease of infarct volume and edema volume, and improved motion function in siMEG3-injected MACO mice. Furthermore, the functional interaction of MEG3 and miR-181b is reflected in experienced pcDNA-MEG3 and miR-181b co-transfected HT22 cell. We observed that miR-181b effectively reverses pro-apoptosis action of MEG3 in HT22 cells and this data suggested the possible neuroprotective action of miR-181b. This contradictory effect of miR-181b with previous study (Peng et al., 2013) may be by reason of experiments with different neural cell lines and distinction of target gene for miR-181b.

These interactions were shown to be crucial for global processes such as gene activation, as well as local events such as the abnormal gene phenotype in response to hypoxia injury. Interestingly, our data support this by showing that 12/15-LOX is the responsive functional protein for MEG3/miR-181b. Recent studies showed that 12/15-LOX upregulation promotes neuronal death under post-hypoxia. Our data also support this fact and showed the time course of 12/15-LOX upregulation in infarction area of MCAO mice. 12/15-LOX is a key enzyme of unsaturated fatty acid metabolism and plays a significant role in the development of cerebral disease, such as Alzheimer's disease and Parkinson's disease (Praticò et al., 2004; Chou et al., 2014). In particular, the facilitating function of 12/15-LOX in pathological process of cerebral ischemic injury has established in ischemic HT22 neuronal cells and in transient focal ischemic brain of rat (Jung et al., 2015; Sun et al., 2015). In addition, our data showed that the physical abnormal 12/15-LOX expression is positively affected by genetic silencing or activation of lncRNA MEG3, whereas negatively regulated by aberrant expression of miR-181b. 12/15-LOX also serves as a target gene for miR-181b and its expression is inhibited via gene being integrated by miR-181b as shown in Figure 4A. These data suggested that 12/15-LOX serves as a final responder for MEG3/miR-181b and mediates hypoxia-induced apoptosis of neuron. We also found that 12/15-LOX overexpression abrogated anti-apoptosis action of miR-181b overexpression (data not shown).

In summary, our present study is for the first time to demonstrate that lncRNA MEG3 functions as a competing endogenous RNA for miR-181b and this interaction plays an important role in ischemia-caused neuronal cell apoptosis. Meanwhile, during cerebral pathological injury, 12/15-LOX is the responsive protein for this lncRNA MEG3/miR-181b axis.

## Author contributions

LH performed the experiments and YG analyzed the data. WH and XLiu contributed reagents/materials/analysis tools. XLv wrote the manuscript. JT conceived and designed the experiments and analyzed the data. All authors read and approved the final manuscript.

### Conflict of interest statement

The authors declare that the research was conducted in the absence of any commercial or financial relationships that could be construed as a potential conflict of interest.
